# Neutrophil-to-lymphocyte ratio in malignant pleural fluid: Prognostic significance

**DOI:** 10.1371/journal.pone.0250628

**Published:** 2021-04-26

**Authors:** Natalia Popowicz, Hui Min Cheah, Cynthia Gregory, Alina Miranda, Ian M. Dick, Y. C. Gary Lee, Jenette Creaney

**Affiliations:** 1 Pharmacy Department, Sir Charles Gairdner Hospital, Perth, Australia; 2 Institute for Respiratory Health, University of Western Australia, Perth, Australia; 3 National Centre for Asbestos Related Disease, Health and Medical Science, University of Western Australia, Perth, Australia; 4 Department of Respiratory Medicine, Sir Charles Gairdner Hospital, Perth, Australia; Medical University of Graz, AUSTRIA

## Abstract

Predicting survival of patients with malignant pleural effusions (MPEs) is notoriously difficult. A robust prognostic marker can guide clinical decision making. The neutrophil-to-lymphocyte ratio (NLR) in blood has been shown to predict survival in many cancers. Pleural fluid bathes the malignant pleural tissues, thus the NLR of the pleural fluid may reflect more closely the local tumour environment. The objective of this study was to explore the prognostic significance of pleural effusion NLR for MPE. We analysed matched effusion and blood from 117 patients with malignant and 24 with benign pleural effusions. Those who had received recent chemotherapy or had a pleurodesis were excluded. Neutrophil and lymphocyte counts in effusions were performed by manual review of cytospin cell preparations by trained observers. Clinical data were extracted from a state-wide hospital database. We found significantly fewer neutrophils (expressed as percentage of total leukocyte count) in pleural fluid than in corresponding blood (9% vs 73%; p<0.001). The NLR was an order of magnitude lower in pleural fluid than in corresponding blood: median [IQR] = 0.20 [0.04–1.18] vs 4.9 [3.0–8.3], p<0.001. Correlation between blood and pleural fluid NLR in MPE patients was moderate (r_s_ = 0.321, p<0.001). In univariate analysis, NLR (>0.745)) in malignant pleural fluid was predictive of poorer survival (HR = 1.698 [1.0054–2.736]; p = 0.030), and remained significant after adjustment for age, sex, presence of a chest drain, cancer type, concurrent infection and subsequent treatment with chemotherapy (HR = 1.786 [1.089–2.928]; p = 0.022). Patients with pleural fluid NLR > 0.745 had a significantly shorter median survival of 130 (95% CI 0–282) days compared to 312 (95% CI 195–428) days for pleural NLR < 0.745, p = 0.026. The NLR in blood was also predictive of poorer survival in MPE patients (HR = 1.959 [1.019–3.096]; p<0.001). The proportion of neutrophils in pleural fluid was predictive of prognosis more strongly than lymphocytes. This study provides evidence that NLR in malignant effusions can predict survival, and therefore may provide prognostic information for this cohort. This prognostic association in the fluid is driven by the presence of neutrophils.

## Introduction

Malignant pleural effusion (MPE) often indicates advanced, disseminated malignancy. MPE most commonly develops due to lung, breast, gastrointestinal and gynaecological cancers as well as malignant mesothelioma (MM). Shortness of breath from the underlying pleural effusion is the most common presenting symptom and is often relieved by pleural fluid drainage. Prognosis of patients with MPE can range from 1 to 18 months and can be dependent on the underlying aetiology and cancer staging [1, 2]. Predicting survival is often difficult and inaccurate, however it remains important for clinicians to enable appropriate treatment planning and for patients so that they can plan their remaining (often limited) lifespan.

Symptom control is the primary goal of MPE management and can be achieved through a number of invasive procedures. Treatment choices including chemotherapy or effusion control strategy, may be guided by prognosis. These procedures may not be appropriate for patients with a poor prognosis where palliative approaches and thoracentesis can provide transient relief of symptoms in the short-term whilst minimising discomfort. Stratifying patients with MPE based on survival is therefore essential to facilitate appropriate treatment choices.

Most of the research efforts to date on prognostication of MPE patients have focussed on clinical (e.g. performance status [1, 3] and tumour type [1] or blood parameters (e.g. including serum neutrophil-to-lymphocyte ratio). Although some pleural fluid markers, e.g. lactate dehydrogenase [1] and pH [4], have been shown to have value, they are generally weak predictors of survival.

The neutrophil-to-lymphocyte ratio (NLR) in blood is a recognized surrogate marker of inflammation and immune response. An elevated blood NLR is associated with poor survival in MPE [1, 5] and its associated cancers including MM and non-small cell lung cancer at various cut-off values ranging from 3 to 9 [6–12], with a threshold ratio of 5 most frequently reported [6–9, 13].

All patients with MPE have pleural fluid sampled for cytological analysis and therefore the NLR is easily available. Examining pleural fluid NLR may provide insight into whether the prognostic power of blood NLR is a reflection of the tumour immune milieu or systemic events. The objective of this study was to explore the prognostic significance of pleural effusion NLR for MPE. A recent study in patients with non-small cell lung cancer (NSCLC) and epidermal growth factor receptor (EGFR) wild-type patients found that pleural NLR was predictive of survival [14], although not for EGFR mutation-positive NSCLC. The present study describes the pleural fluid NLR compared with blood NLR in patients with MPE with various underlying aetiologies and provides evidence that pleural NLR is predictive of survival.

## Materials and methods

Pleural fluid samples of patients attending Sir Charles Gairdner Hospital in Perth, Western Australia were prospectively collected between January 2010 and December 2015 from patients who provided informed written consent. Inclusion criteria included the presence of a pleural effusion requiring drainage; exclusion criteria included concomitant immunosuppressive medication usage, age less than 18 years, pregnancy and inability to provide informed consent. This study was approved by the Sir Charles Gairdner and Osborne Park Health Care Group Human Research Ethics committee. Samples were collected in sterile containers following pleural drainage and immediately transferred at ambient temperature to the laboratory. Samples with >100mL and with > 5 x 10^6^ total cells that were processed immediately were included. Samples were centrifuged at 1000 *g* for 10 minutes and re-suspended as a single cell suspension in RPMI-1640 media (Invitrogen) and 10% foetal calf serum (Invitrogen) and centrifuged onto a microscope slide using a StatSpin Cytofuge 2 (Beckman Coulter) following the manufacturer’s instructions. Cytospins were stained using Rapid Stain (Amber Scientific) and 400 cells per slide were manually reviewed and classified as tumour, mesothelial, macrophage, lymphocyte, neutrophil, eosinophil or other by a cytopathology technician (AM) and an experienced trained researcher (CG). Cronbach’s alpha test of internal consistency confirmed excellent inter-observer variability with above 0.900 scored for 4 of 5 categories and 0.844 for the remaining one.

The hospital databases were interrogated for clinical (including age, gender, treatment, concurrent or history of pleural infection and survival) and laboratory data of individual patients. In particular, blood total (and differential) leucocyte counts performed within 24 hours of the collected pleural sample were also identified. Chemotherapy treatment data were confirmed through pharmacy dispensing and medical records. Survival data were censored on 16 August 2016.

Samples were classified based on pathological, biochemical and clinical review as malignant or benign pleural effusions. Malignant effusions all had histo-/cyto-logical and ancillary immunostaining confirming cancer cells were present in the pleural fluid or tissue biopsy, and/or were an effusion in a patient with known metastatic cancer where there were no alternative fluid aetiologies. MPE were further classified as being related to MM or other cancers. Benign effusions were classed as exudates or transudates by Light’s criteria [15] and confirmed to be unrelated to malignancy during follow-up (median 222; IQR 78–589 days). Samples with diagnostic uncertainties were excluded.

Where multiple pleural samples were available from an individual patient, the first available sample according to collection date with a matching blood leukocyte count that met inclusion and exclusion criteria was included. Samples were excluded if the patient received recent chemotherapy as this may affect leukocyte counts. Exclusion times from chemotherapy were based on standard nadir recovery ranges for each specific chemotherapy agent [16, 17]. Samples were also excluded if patients had undergone a pleurodesis (e.g. with talc) within 3 months of fluid collection as this may impact neutrophil migration. Samples collected via an established chest tube or indwelling pleural catheter (IPC) were noted and analysed as a subgroup.

NLR was calculated by dividing the absolute neutrophil and lymphocyte counts obtained in blood or pleural fluid. The percentage of neutrophils and lymphocytes in blood and pleural fluid was calculated from the total leukocyte count.

Data were analysed using SigmaPlot 12.5 (Systat Software, San Jose, CA), and presented as mean (±SD) or median [IQR] based on normality of data. The Wilcoxon Signed-Rank test and paired t-test were used for analyses of non-parametric and parametric data respectively. The Pearson and Spearman Rank Correlations (for parametric and non-parametric data respectively) were used to determine any relationship between serum and pleural fluid NLR. Survival analysis was performed using Cox proportional hazards regression and data reported with 95% confidence intervals. Median survival times between groups was calculated from the Kaplan Meier survival estimates, using SPSS (v.22, Armonk, NY). For the purpose of dichotomizing NLR and cell percentage variables into groups, Cutoff Finder (http://molpath.charite.de/cutoff/) was used, to determine the value that optimized the log-rank test significance between the two groups. Significance was defined as p<0.05.

A power calculation was performed for a potential analysis of two equal size groups of high and low values of pleural effusion NLR, based upon an assumption that the true HR is 1.81 as reported in the published meta-analysis of the prognostic role of dichotomised NLR in solid tumours [12]. Given a conservative median overall survival for patients with malignant pleural effusions of 12 months, we estimate that a cohort of 90 patients in total will provide a power of 0.8 at the p< 0.05 level, to test the hypothesis that a higher pleural NLR is associated with a reduced survival.

## Results

### Sample and patient characteristics

**Fig 1:**
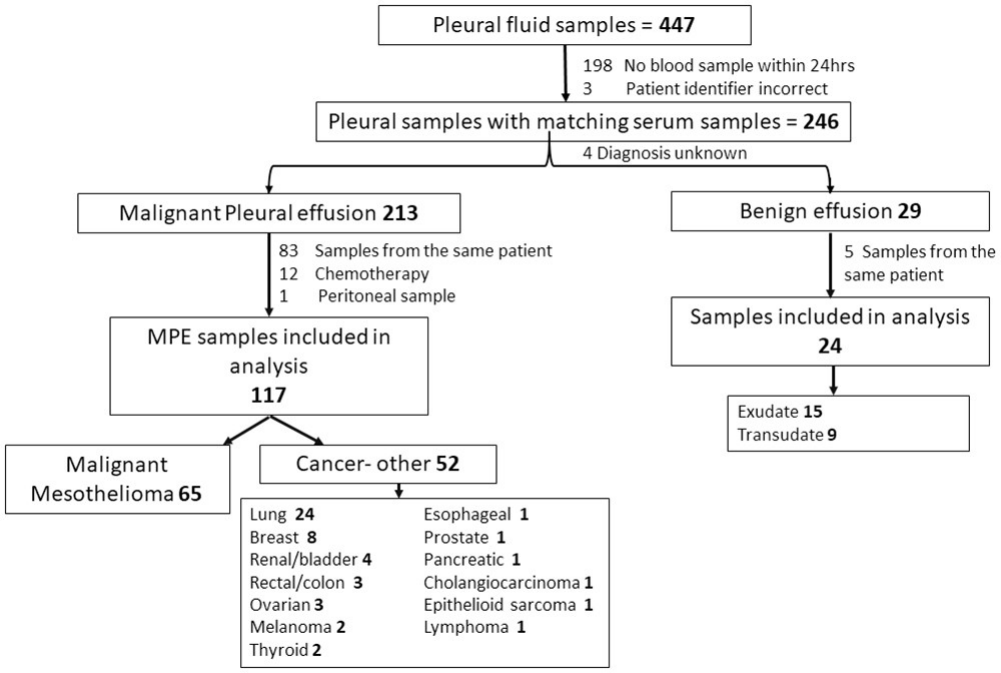
Flow diagram of samples included in analysis.

A total of 447 pleural fluid samples were identified during the study period. Of these 246 samples had matching blood leukocyte counts performed within 24 hours of pleural sample collection. Pathologically confirmed diagnosis was possible in the majority (98.4%) of cases, with 213 MPE and 29 benign effusions identified. Multiple samples available from the same individual (n = 43) resulted in 89 samples being excluded with a further 12 samples excluded due to recent chemotherapy and 1 sample excluded as it was identified as a peritoneal (not pleural) effusion (Fig 1). No samples were excluded due to treatment with a sclerosing agent. A total of 35 patients had an IPC or chest drain *in situ* at the time of collection; 17 with MM, 16 with other malignancy and 2 with benign effusion. Twenty-three patients had a concurrent infection at the time of effusion; 17 malignant and 6 with benign effusion (S1 Table).

In total 141 paired blood and pleural fluid samples (117 MPE and 24 benign effusions) formed the analysis set. Most patients (84%) were male and 68 (±13) years of age. MM was the cause of MPE in over half (n = 65, 55%) of cases. Samples were collected at a median of 161 (22–459) days following diagnosis of malignancy in the MPE cohort (S1 Table).

At census, 77% (n = 90) of patients with MPE (79% of the non-MM and 75% of the MM cohort) were deceased. The median survival time for patients with MPE from sample collection was 285 (IQR 216–814) days. Survival of MM and other cancer cohorts are presented in Table 1. MM patients had a better prognosis from the time of MPE sample collection than patients with other cancer types (HR = 0.55, 0.36–0.84; p = 0.006).

**Table 1:** Survival from sample collection in MPE cohorts.

Cohort descriptor	n	Survival from sample date Median (IQR) days
**All—Malignant Pleural Effusion**	**117**	**284 (92–620)**
MM	65	396 (216–814)
Other cancers	52	126 (39–334)
*Lung cancer*	*24*	*121 (39–284)*
*Breast cancer*	*8*	*167 (75–475)*

MM, malignant mesothelioma; IQR, interquartile range.

### Neutrophil and lymphocyte percentages differ between pleural fluid and blood

**Fig 2:**
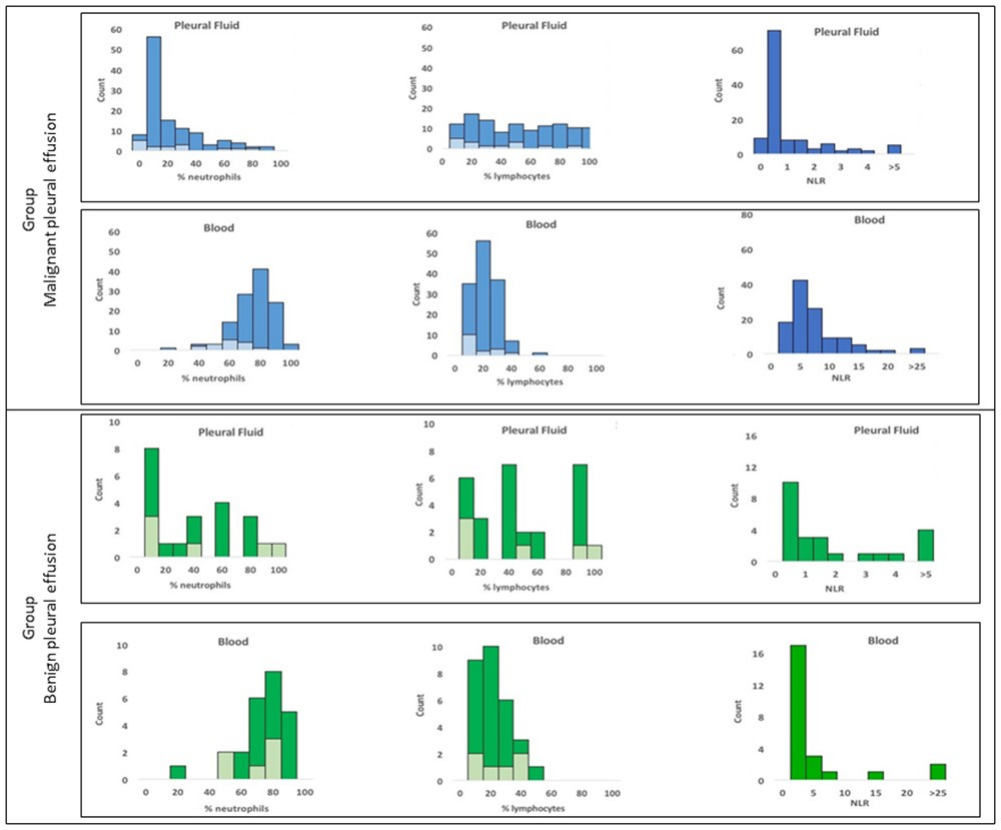
Frequency distribution of samples by % neutrophils, % lymphocytes and Neutrophil to Lymphocyte Ratio (NLR) in malignant pleural effusions (left panel) and blood (right panel). Results from patients with malignant pleural effusions (MPE) are in blue and benign effusions in green. Samples with concurrent infection are shown in lighter colour for neutrophil and lymphocyte distribution.

Neutrophils, presented as a percentage of total leucocytes, were significantly lower in the pleural fluid (median 9%, IQR 2–33) than in blood (73%, 65–80), p<0.001 (Table 2). Conversely, the overall proportion of lymphocytes was significantly greater in pleural fluid (41%, 20–73) relative to blood (14%, 9–22), p<0.001 (Fig 2). A low correlation between the percentage of neutrophils (r_s_ = 0.232, p = 0.006) and lymphocytes (r = 0.129, p = 0.134) in blood and pleural fluid was observed.

**Table 2:** Percentage of neutrophils and lymphocytes in pleural fluid and in blood.

Category	All samples (n = 141) Median (IQR)	Malignant (n = 117) Median (IQR)	Benign (n = 24) Median (IQR)	*p value*
				*malignant vs benign*
% neutrophils (pleural)	**9 (2–33)**	8 (2–24)	33 (3–61)	*p = 0*.*019*
% neutrophils (blood)	**73 (65–80)**	74 (66–80)	71 (63–77)	*p = 0*.*422*
*p value (pleural vs blood)*	*p<0*.*001*			
% lymphocytes (pleural)	**41 (20–73)**	45 (20–72)	39 (19–84)	*p = 0*.*767*
% lymphocytes (blood)	**14 (9–22)**	14 (9–22)	16 (9–21)	*p = 0*.*60*
*p value (pleural vs blood)*	*p<0*.*001*			

IQR—interquartile range.

Benign pleural effusions (33% [3–61]) had a significantly higher proportion of neutrophils than MPE (8% [2–24], p = 0.019). The proportion of lymphocytes between benign and malignant effusions was not statistically significantly different (39% [19–84] vs 45% [20–72], p = 0.767). There were no differences observed between blood neutrophil or lymphocyte proportions between benign and malignant groups (Table 2).

### Effect of chest drains and infection on neutrophil percentages

For patients with MPE, the percentage of neutrophils in pleural fluid was not affected by the presence, at the time of pleural fluid sampling, of a chest tube or IPC (9.0% [IQR 2.1–27.5] compared to 7.7% [2–24] for non-IPC/chest drain, p = 0.85).

The percentage of neutrophils in blood was not different in patients with MPE with (n = 17) and without (n = 101) concurrent infection (76% [62–84] vs 73% [66–80], p = 0.446) although there was a significant difference in pleural fluid neutrophil percentages (19% [7–36] vs 6% [2–22], p = 0.049).

### Neutrophil-to-lymphocyte ratio in blood and pleural fluid significantly differ

**Fig 3:**
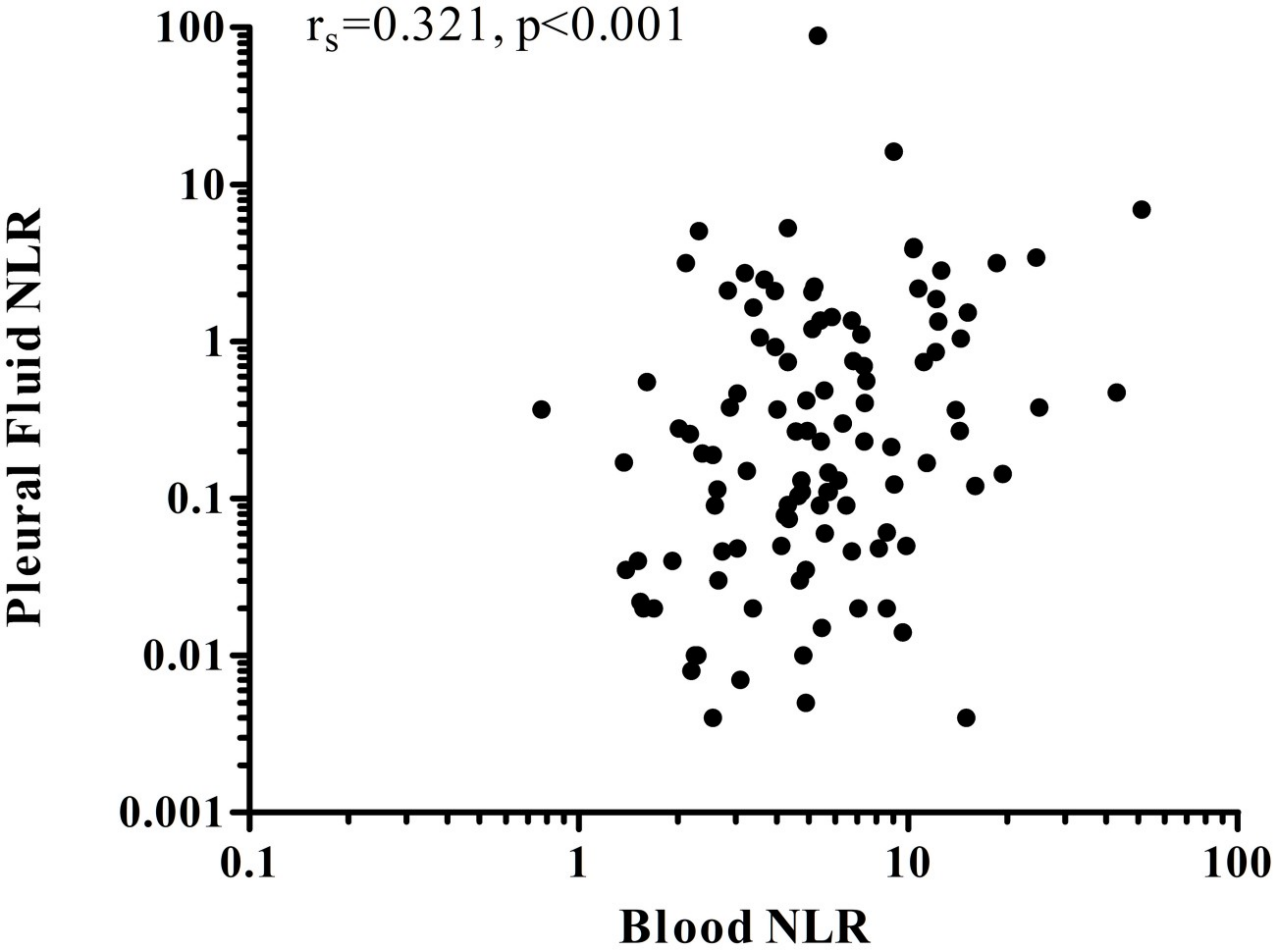
Correlation between blood and pleural fluid NLR in patients with MPE.

The median NLR in pleural effusions was an order of magnitude lower than in blood (0.20 [IQR 0.04–1.18] vs 4.9 [3.0–8.3], p<0.001). Pleural NLR was lower in malignant effusions than in benign ones (0.17 [0.04–0.91] vs 0.744 [0.05–3.19], p = 0.056). MPE patients with a chest tube or IPC had comparable NLR levels to those without (0.13 [0.03–0.51] vs 0.21 [0.04–1.08], p = 0.461). Correlation between blood and pleural fluid NLR in MPE patients was moderate (r_s_ = 0.321, p <0.001) (Fig 3).

### Neutrophil-to-lymphocyte ratio as a prognostic marker

**Fig 4:**
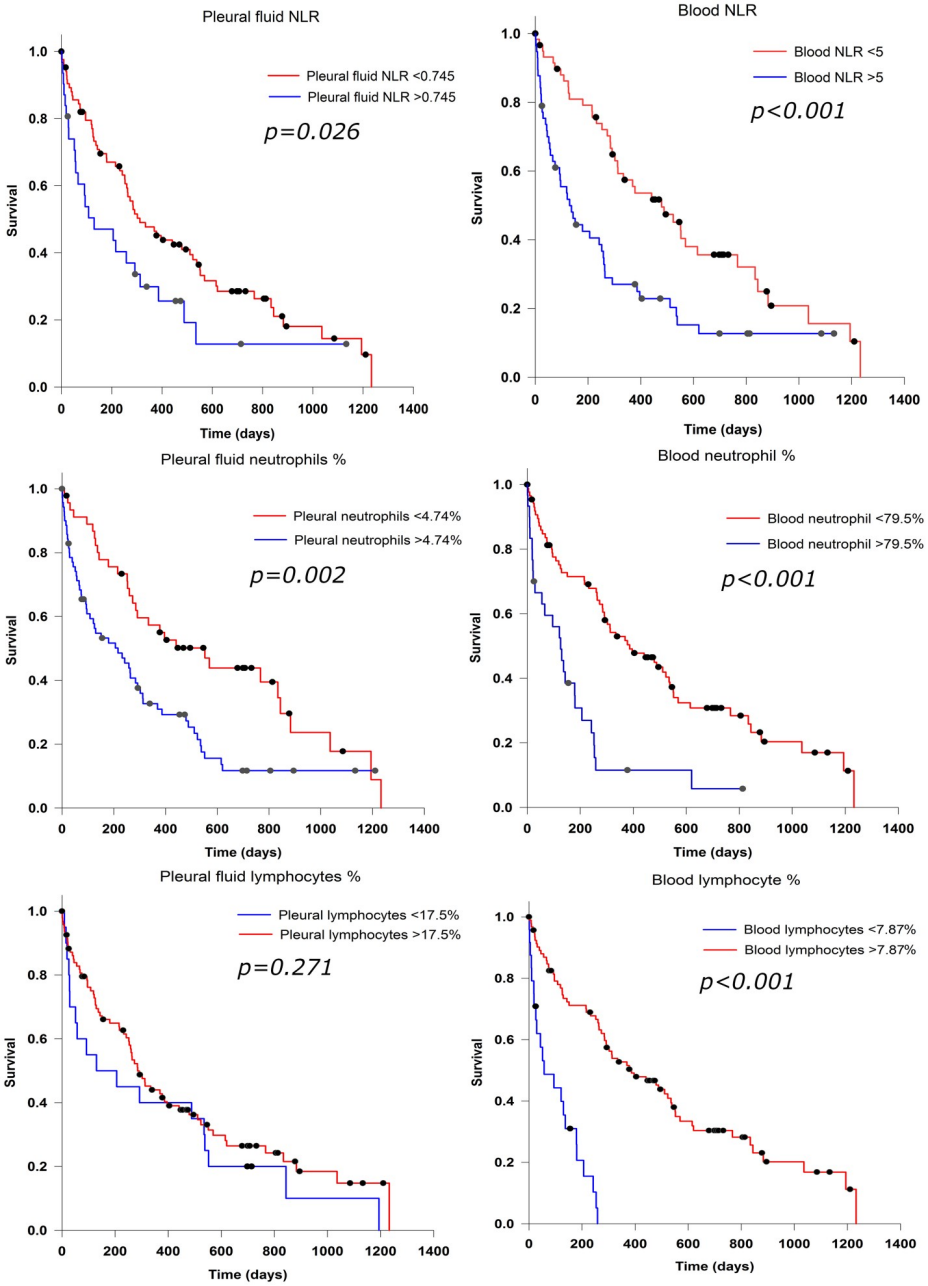
Survival analysis in patients with MPE.

The NLR within pleural fluid of patients with MPE was identified as an individual predictor of overall survival in a univariate analysis (HR 1.7, [95% CI 1.054–2.736], p = 0.030), Table 3. In the subgroup of patients with metastatic cancer, pleural NLR was predictive of survival (HR 2.25, [1.102–4.588], p = 0.026) and a similar trend was observed in the MM subgroup (HR 1.85, [0.945–3.609], p = 0.073), indicating that MM was not a significant modifier of this relationship. A pleural fluid NLR > 0.745 was associated with significantly shorter median survival 130 (95% CI 0–282) days compared to 312 (95% CI 195–428) days for pleural NLR < 0.745, p = 0.026 (Fig 4). After adjusting for age, sex, chest drain, cancer type, concurrent infection and treatment with chemotherapy the association between pleural NLR and survival for patients with MPE was no longer significant when analysed as a continuous measurement (HR 1.018, 95% CI 0.996–1.041, p = 0.115), but remained so in the grouped analysis comparing high with low pleural NLR (HR 1.786, 95% CI 1.089–2.928, p = 0.022).

**Table 3:** Survival analysis for patients with MPE.

Descriptor	Univariate HR	*p*	Multivariate HR^a^	*p*
Age (per 10 year increase)	1.022 (0.845–1.24)	0.835		
Sex (F v M)	0.740 (0.476–1.152)	0.182		
Chest drain (yes v no)	0.947 (0.58–1.528)	0.823		
Cancer type (MM v other cancer)	0.546 (0.356–0.838)	**0.006**		
**NLR**				
Blood (per unit increase)	1.047 (1.025–1.069)	**<0.001**	1.044 (1.019–1.070)	**<0.001**
Blood (high v low; cut-off = 5)	2.046 (1.338–3.130)	**0.001**	1.959 (1.240–3.096)	**0.004**
Effusion (per unit increase)	1.026 (1.004–1.048)	**0.018**	1.018 (0.996–1.041)	0.1115
Effusion (high v low; cutoff = 0.745)	1.698 (1.054–2.736)	**0.030**	1.786 (1.089–2.928)	**0.022**
**% neutrophils**				
Blood (per unit increase)	1.043 (1.020–1.066)	**<0.001**	1.042 (1.019–1.067)	**<0.001**
Blood (high v low; cutoff = 79.5)	2.958 (1.821–4.803)	**<0.001**	2.970 (1.797–4.910)	**<0.001**
Effusion (per unit increase)	1.012 (1.001–1.022)	**0.031**	1.011 (1.000–1.022)	**0.045**
Effusion (high v low; cutoff = 4.74)	2.014 (1.288–3.148)	**0.002**	2.290 (1.423–3.685)	**<0.001**
**% lymphocytes**				
Blood (per unit increase)	0.94 (0.913–0.968)	**<0.001**	0.942 (0.914–0.971)	**<0.001**
Blood (high v low; cutoff = 7.87)	0.181 (0.102–0.319)	**<0.001**	0.208 (0.114–0.381)	**<0.001**
Effusion (per unit increase)	1.00 (0.992–1.007)	0.956	0.995 (0.986–1.005)	0.337
Effusion (high v low; cutoff = 17.5)	0.743 (0.440–1.253)	0.265	0.525 (0.287–0.961)	**0.037**

HR—Hazard Ratio (with 95% confidence intervals).

^a^ Multivariate analysis are for the NLR PE and blood variables, and the %neutrophils PE and blood variables, adjusted together with age, sex, chest drain, cancer type, concurrent infection and treatment with chemotherapy as indicated in the table.

For patients with MPE, the NLR in the blood as a continuous variable, was predictive of survival (HR 1.05 [95% CI 1.02–1.07]; p<0.001). Patients with blood NLR ≥ 5 had a median survival of 130 (95% CI 64–195) days from sample collection compared to 479 (95% CI 289–669) days for NLR < 5, p<0.001 (Fig 4). Multivariate analysis for blood NLR > 5 showed significantly poorer overall survival: HR 2.0 (95% CI 1.3–3.1), p = 0.002 (Table 3).

### Neutrophils as a prognostic marker in effusion

Survival analysis revealed that per unit increase, neutrophil percentages within the blood and fluid were significant prognostic factors (Table 3). The proportion of neutrophils in MPE was not normally distributed (Fig 2). For survival analysis an optimal threshold of 4.74% for the percentage of neutrophils in MPE was determined, with a high percentage of neutrophils (>4.74%) in the effusions as a proportion of total leucocyte count associated with poorer median survival of 216 (95% CI 88–344) days compared to 552 (95% CI 293–810) days for patients in the low neutrophil group, p = 0.002. Neutrophil proportions in both pleural fluid (optimised cut-off 4.74%) and blood (79.5%) predicted prognosis with HR of 2.290 (1.423–3.685) and 2.970 (1.797–4.910) respectively, independently of tumour type, age, sex, chest-drain status, concurrent infection and treatment with chemotherapy.

### Lymphocytes are not a prognostic marker in effusion

The proportion of lymphocytes in MPE was evenly distributed (Fig 2). No association with the percentage of lymphocytes in effusions and survival was observed for continuous or dichotomised values in univariate analysis, although a weak association was found in multivariate analysis (Table 3). This is contrary to lymphocyte percentage in blood, where patients with a high percentage of lymphocytes had significantly longer median survival compared those with low lymphocyte percentage: 386 days (95% CI 222–550) vs 57 days (95% CI 0–135), p<0.001 (Fig 4).

## Discussion

This study of pleural fluid NLR finds that the proportion and distribution of neutrophils and lymphocytes differed considerably between the blood and pleural fluid suggesting that the pleural immune/inflammatory responses are, in part, compartmentalized. In this hypothesis generating study, the pleural fluid NLR predicted prognosis. The percentage of neutrophils within the pleural fluid, but not the lymphocytes, was associated with poor survival, suggesting that in the effusion NLR is driven by neutrophils.

Systemic NLR has been validated as a prognostic biomarker in many cancers [12, 18]. Our study confirmed the prognostic value of blood NLR, with similar values as reported in previous studies of MPEs [1, 19–21]. The balance of neutrophils and lymphocytes in the blood has been suggested to reflect both the burden and aggressiveness of the tumour, (as NLR increase with tumour stage, nodal status and disease extent [18]) as well as being a surrogate marker of the tumour-host interaction, whereby a relatively straightforward and easy to obtain semi-quantification of the interplay between the pro-tumour neutrophil and the anti-tumour lymphocyte cellular compartments can be determined.

MPEs develop at the site of the pleural tumour therefore the NLR within the effusion itself allows assessment of the immunological cellular milieu in the malignant environment. There was a weak, but significant, monotonic relationship between blood and effusion NLR, as well as distinctly different levels in the two compartments, and our study supports that NLR in effusions may have prognostic value. There was an inverse linear relationship between effusion NLR and survival which suggests a dose-related effect. However, the dichotomised threshold value for the NLR used in this study will need to be validated and optimized in larger future studies.

NLR as a biomarker, reflects overall immunological status. In blood we noted that as independent variables both the higher percentage of neutrophils and the lower the percentage of lymphocytes were predictive of poor survival confirming findings from previous studies in multiple tumour types including prostate, nasopharyngeal, gastric, renal, lung and NSCLC [22–26]. In effusions, however, only the percentage of neutrophils, not lymphocytes, was prognostic, with higher neutrophil counts associated with poorer survival. This suggests that it is the increased neutrophils rather than decreased lymphocytes that drives the predictive value of pleural fluid NLR. Neutrophils may play a role in tumorigenesis as they can secrete reactive oxygen species causing DNA damage. Their contribution to prognosis may be more related to increased levels of some cytokines they secrete, specifically vascular endothelial growth factor and interleukins including IL-6 and IL-8 known to be associated with angiogenesis promoting tumour growth, along with MPE formation [27–31]. Neutrophils, along with other cells of the myeloid lineage, have also been shown to suppress T cell function [32]. At the tumour site the ratio of specific lymphocyte sub-populations such as T effector to T regulatory cells may have greater prognostic significance than total lymphocyte numbers.

The limitation of this study is the lack of validation in an independent cohort. Furthermore, our cohort had a high proportion of patients with MM due to local prevalence. However systemic NLR has been shown to reflect prognosis of MM as it does for common metastatic pleural cancers that cause MPE. Also, patients on recent chemotherapy were excluded, as this can induce acute neutropenia and may not be reflective of the ongoing tumour-immune interaction; however, excluding these patients may not reflect real life situations of sampling. Despite the heterogeneity of the cohort we confirmed previous findings that blood NLR was an independent predictor of prognosis, which supports the validity of the cohort. A strength of our study was that samples were included from any time point following diagnosis (median 182 days) and therefore may have greater clinical relevance as prognostic markers.

This study describes the neutrophil and lymphocyte distributions in MPEs from various aetiologies and found a prognostic value of pleural fluid NLR and of pleural fluid neutrophils (as a percentage of total leukocyte counts). Further studies are needed to determine whether pleural fluid NLR may be a simple and useful biomarker for predicting prognosis in patients with malignant effusions.

## Supporting information

S1 Table (XLSX)
